# Photoplethysmography and intracardiac pressures: early insights from a pilot study

**DOI:** 10.1093/ehjdh/ztae020

**Published:** 2024-03-07

**Authors:** Niels T B Scholte, Annemiek E van Ravensberg, Roos Edgar, Antoon J M van den Enden, Nicolas M D A van Mieghem, Jasper J Brugts, Judith L Bonnes, Nico Bruining, Robert M A van der Boon

**Affiliations:** Department of Cardiology, Cardiovascular Institute, Erasmus Medical Center, Dr. Molewaterplein 40, 3015 GD, Rotterdam, the Netherlands; Department of Cardiology, Cardiovascular Institute, Erasmus Medical Center, Dr. Molewaterplein 40, 3015 GD, Rotterdam, the Netherlands; Department of Cardiology, Radboud University Medical Center, Nijmegen, the Netherlands; Department of Cardiology, Cardiovascular Institute, Erasmus Medical Center, Dr. Molewaterplein 40, 3015 GD, Rotterdam, the Netherlands; Department of Cardiology, Cardiovascular Institute, Erasmus Medical Center, Dr. Molewaterplein 40, 3015 GD, Rotterdam, the Netherlands; Department of Cardiology, Cardiovascular Institute, Erasmus Medical Center, Dr. Molewaterplein 40, 3015 GD, Rotterdam, the Netherlands; Department of Cardiology, Radboud University Medical Center, Nijmegen, the Netherlands; Department of Cardiology, Cardiovascular Institute, Erasmus Medical Center, Dr. Molewaterplein 40, 3015 GD, Rotterdam, the Netherlands; Department of Cardiology, Cardiovascular Institute, Erasmus Medical Center, Dr. Molewaterplein 40, 3015 GD, Rotterdam, the Netherlands

**Keywords:** Heart failure, Wearables, Intracardiac Pressures, Telemedicine, Photoplethysmography

## Abstract

**Aims:**

Invasive haemodynamic monitoring of heart failure (HF) is used to detect deterioration in an early phase thereby preventing hospitalizations. However, this invasive approach is costly and presently lacks widespread accessibility. Hence, there is a pressing need to identify an alternative non-invasive method that is reliable and more readily available. In this pilot study, we investigated the relation between wrist-derived photoplethysmography (PPG) signals and the invasively measured pulmonary capillary wedge pressure (PCWP).

**Methods and results:**

Fourteen patients with aortic valve stenosis who underwent transcatheter aortic valve replacement with concomitant right heart catheterization and PPG measurements were included. Six unique features of the PPG signals [heart rate, heart rate variability, systolic amplitude (SA), diastolic amplitude, crest time (CT), and large artery stiffness index (LASI)] were extracted. These features were used to estimate the continuous PCWP values and the categorized PCWP (low < 12 mmHg vs. high ≥ 12 mmHg). All PPG features resulted in regression models that showed low correlations with the invasively measured PCWP. Classification models resulted in higher performances: the model based on the SA and the model based on the LASI both resulted in an area under the curve (AUC) of 0.86 and the model based on the CT resulted in an AUC of 0.72.

**Conclusion:**

These results demonstrate the capability to non-invasively classify patients into clinically meaningful categories of PCWP using PPG signals from a wrist-worn wearable device. To enhance and fully explore its potential, the relationship between PPG and PCWP should be further investigated in a larger cohort of HF patients.

## Introduction

Heart failure (HF) is a prevalent and challenging chronic condition affecting over 64 million people globally, with its incidence expected to rise due to an aging population.^1^ Despite significant medical advances, HF still carries high morbidity and mortality rates, and is associated with frequent outpatient visits and hospitalizations. This places a substantial burden on healthcare systems, emphasizing the need for effective strategies to reduce readmissions and enhance outpatient care. Remote monitoring (RM) technologies have emerged as a solution, enabling early detection of HF deterioration and proactive treatment to avert hospitalizations. A recent comprehensive meta-analysis demonstrated the efficacy of both invasive and non-invasive RM in lowering all-cause mortality and HF-related hospitalizations.^2^ Non-invasive RM primarily addresses symptomatic HF, whereas invasive approaches, relying on haemodynamic parameters including the pulmonary capillary wedge pressure (PCWP), can detect deterioration even before symptoms manifest.^3^ This offers the advantage of a more extended time window for intervening in HF deterioration, however, cost constraints and healthcare system limitations restrict its use. To expand the accessibility of haemodynamic monitoring, it is essential to explore non-invasive techniques that can potentially correlate with haemodynamic pressures, specifically the PCWP. Photoplethysmography (PPG) is commonly employed in both consumer-grade and medical-grade wearable devices for monitoring physiological parameters. If PPG signals can accurately approximate PCWP, wearable devices could offer opportunities for RM of HF patients. Hence, the objective of this prospective pilot trial was to investigate whether PPG signal characteristics could be correlated with invasively measured PCWP.

## Methods

In this single-centre, prospective, observational study, we included patients with aortic stenosis who underwent transcatheter aortic valve replacement (TAVR) and participated in both the DETECT-I and PLUTO-II studies.^4^ In brief, the objective of the DETECT-I study is to develop an algorithm for circulatory arrest detection using PPG data collected during TAVR, utilizing a medical-grade wrist-worn PPG device (CardioWatch Bracelet 287-2, Corsano Health B.V.). In the PLUTO-II study, pressure–volume (PV) relationships were studied before and after TAVR by means of *in vivo*, ventricular conductance catheter measurements. For mandatory volume calibration, all patients underwent right heart catheterization (RHC) allowing determination of cardiac output based on thermodilution as well as PCWP measurements. More elaborate information of the PLUTO-II study can be found in previously published work.^4^ Combining data from both studies allowed for the distillation of PPG signals and PCWP measurements during TAVR.

### Data collection

For baseline characteristics, we collected demographic characteristics, medical history, and haemodynamic and echocardiographic parameters from our local electronic health record. Multi-wavelength PPG signals were continuously recorded and transmitted wirelessly using Bluetooth from the bracelet via the manufacturers’ smartphone application to a secured cloud (Corsano Research Portal). For the analysis, data samples from a single channel with a frequency of 128 Hz and a green light signal (wavelength of 525 nm) were used, as these contain less artefacts. The PCWP was assessed through RHC before valve implantation, which was performed by placing a Merit Medical Criticath® thermodilution catheter into the pulmonary artery by jugular or femoral vein access. Since PCWP can be measured multiple times during a catheterization, only the last recorded value was used.

### Statistical analysis

**Figure 1:**
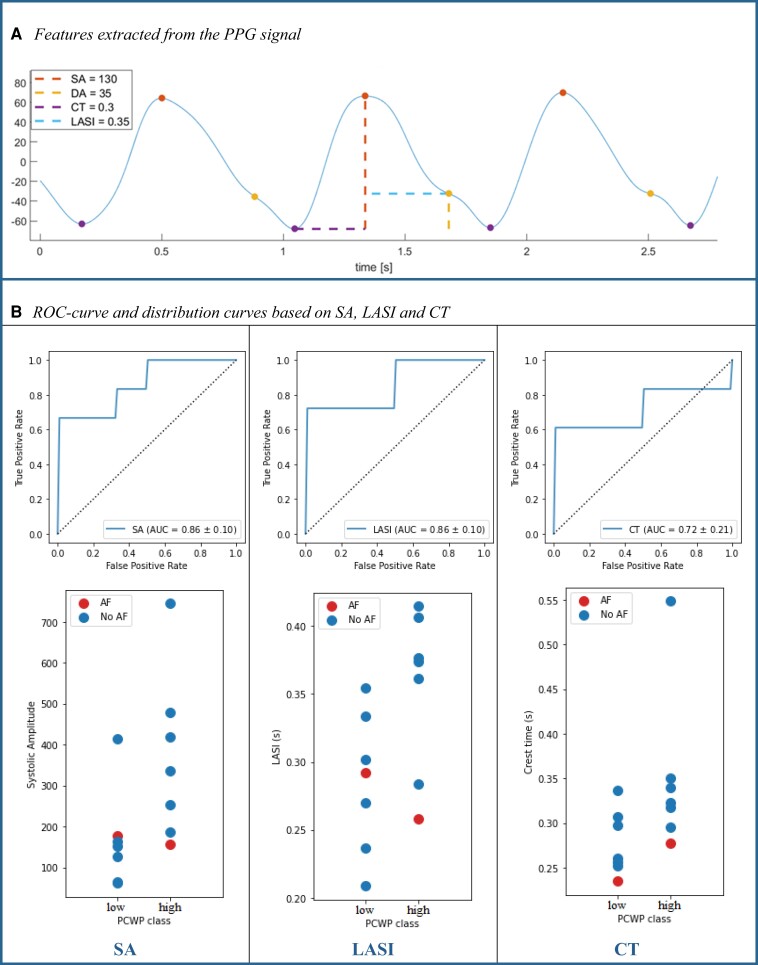
(*A*) Features extracted from the photoplethysmography (PPG) signal: systolic amplitude (SA), diastolic amplitude (DA), crest time (CT), and large artery stiffness index (LASI). Additionally, the distance between detected systolic peaks was used to calculate the heart rate and heart rate variability, (*B*) ROC curve and distribution curve of model based on SA, LASI, and CT, respectively.

The PPG signal in the 70 s interval (10 s filtered leaving 60 s for analysis) preceding the PCWP measurement was selected for each subject. In case of visually identifiable artefacts, the start time of the window was advanced by a maximum of 5 min. Then, the PPG signals were pre-processed and a 2nd order Butterworth band-pass filter with a lower threshold of 1 Hz and an upper threshold of 5 Hz was used to filter out the wandering baseline (low frequencies) and noise (high frequencies). Afterwards, six unique features were extracted: the heart rate (HR), heart rate variability (HRV), systolic amplitude (SA), diastolic amplitude (DA), crest time (CT), and large artery stiffness index (LASI), based on proposed features from literature.^5^ (*Figure 1A*) These features can be extracted from any device providing raw PPG data, not limited to the CardioWatch only. Subsequently, the features were used to estimate the PCWP values. Linear regression and linear discriminant analysis (LDA) were used to estimate the continuous and categorical [<12 mmHg (low) vs. ≥ 12 mmHg (high)] PCWP, respectively.^6^ The models were based on a single feature at a time and validated with three-fold cross-validation. The regression models were evaluated with the resulting R2 value and the classification models with the AUC of the receiver operating characteristic (ROC) curve. Additionally, the two PCWP classes (low vs. high) were statistically compared using a Mann–Whitney *U* test. *P*-values of <0.05 were considered as statistically significant. Bonferroni correction was used to account for multiple testing.

## Results

### Baseline characteristics

A total of 14 patients were included in this pilot study. *Table 1A* shows the baseline characteristics of the included patients. The mean age of the population was 79.3 ± 6.8 years, and 79% were men. Regarding antecedents, 29% of the patients had HF and 14% had a form (paroxysmal, persistent, or permanent) of atrial fibrillation. The median PCWP value was 11.5 mmHg (interquartile range: 5.5–13.5), and 50% of the values were ≥ 12 mmHg (*Table 1B*).

**Table 1:** Patient characteristics, model performances, and feature differences between low and high PCWP

A) Demographics		B) Echocardiographic parameters	
	*n* = 14		*n* = 14
Age, mean ± SD	79.3 ± 6.8	PCWP, median (IQR)	11.5 (5.5–13.5)
Male	11 (78.6)	PCWP ≥ 12 mmHg	7 (50.0)
HF	4 (28.6)	LVEF (%), Mean ± SD	47.4 ± 12.5
Coronary artery disease	6 (42.9)	Moderate/severe AS	14 (100.0)
Hypertension	8 (57.1)	Moderate/severe MR	1 (7.1)
Diabetes mellitus	5 (35.7)		
Atrial fibrillation	2 (14.3)		
History of smoking	6 (42.9)		

C) Regression and classification performance results			
	Correlation with	Regression	Classification
Feature	the PCWP	R2	AUC
Heart rate (/s)	0.13	−1.42	0.36
HRV (ms)	0.09	−2.47	0.50
SA	0.08	−0.79	0.86
DA	0.07	−0.93	0.44
CT (s)	0.06	−2.74	0.72
LASI (s)	0.05	−1.14	0.86

D) PPG features divided by PCWP class (low vs. high)							
	Low PCWP			High PCWP			
Feature	Median	Q1	Q3	Median	Q1	Q3	*P*-value
Heart rate (/s)	73.4	62.7	77.5	71.8	68.8	73.2	1.00
HRV (ms)	87.9	49.1	138.0	33.8	25.6	63.0	0.16
SA	152.7	96.2	170.7	336.3	220.0	449.6	0.02
DA	178.3	134.2	213.4	345.0	237.1	498.7	0.03
CT (s)	0.26	0.25	0.30	0.32	0.31	0.35	0.04
LASI (s)	0.29	0.25	0.32	0.37	0.32	0.39	0.05

SD, standard deviations; PCWP, pulmonary capillary wedge pressure; IQR, interquartile range; LVEF, left ventricular ejection fraction; AS, aortic stenosis; MR, mitral regurgitation; R2, coefficient of determination; AUC, area under the curve; HRV, heart rate variability; SA, systolic amplitude; DA, diastolic amplitude; CT, crest time; LASI, large artery stiffness index.

### Main analyses

All PPG features resulted in regression models with negative R2 values indicating low correlations with invasive PCWP (*Table 1C*). The classification models exhibited better performances. Specifically, the models based on SA and LASI achieved an AUC of 0.86, while the models based on the CT resulted in an AUC of 0.72. The corresponding ROC curves of these features are shown in *Figure 1B, C*, and *D*.

The distribution of the SA and the LASI for each PCWP class (*Figure 1B*) showed that a reasonable distinction can be made between the two classes based on these individual features, which is in line with the AUC scores of their respective models.

Notably, the two patients with atrial fibrillation consistently reside at the periphery of their class distribution, highlighting the distinct morphological differences. Despite the visible difference between the classes for every feature, the SA, LASI, and CT did not significantly differ between the PCWP classes according to the Mann–Whitney tests after Bonferroni correction (significance level *α* = 0.0167) (*Table 1D*).

## Discussion

In this pilot study of patients undergoing TAVR, we were able to distinguish patients with low and high PCWP from relevant features derived from high-quality PPG signals recorded during simultaneous invasive pressure monitoring. Regrettably, it was more challenging to estimate the exact PCWP value using these features. To the best of our knowledge, this is the first study that solely uses PPG signals derived from a wearable to approximate PCWP. In a previous study, PPG was used as component of a multisensor device, including among others PPG and ECG, to estimate intracardiac pressures, which showed an AUC of 0.74 for an PCWP ≥ 22 mmHg.^7^ Instead of using features derived from PPG signals only, this multisensory device uses pulse transit time that requires data from PPG and ECG. In future research, it is important to investigate the currently unknown physiology underlying the relation between PPG derived features and whether combining these features together or other non-invasive parameters adds value to estimating PCWP.

The PCWP is the gold standard measurement of congestion, and its invasive monitoring has become an important factor in modern HF management both for inpatients and outpatients. These techniques convert biological signals into waveforms, forming the foundation for clinical interpretation. Yet, the measurement remains associated with an invasive procedure that is not always readily available for all HF patients. As such, a non-invasive method of estimating the PCWP could contribute to the early detection of worsening HF—an important goal that is associated with reduced mortality and rates of rehospitalization.^8^ Non-invasive measurements of intracardiac pressures have ranged from clinical examination, echocardiographic parameters to venous and arterial pressure wave form analysis with differing results.^9,10^ Especially, the latter two have been increasingly studied over the last years because of the possibility to incorporate this in a simple handheld device used for RM. In recent years, innovation in healthcare has allowed us to utilize new technology, such as wearables, to capture biological signals in a non-invasive manner. Additionally, the utilization of deep learning holds the promise of leveraging these measurements to estimate quantities that are typically acquired with invasive studies.

Our study’s limitations warrant thorough discussion. First, the constrained sample size and lack of statistical power necessitate cautious interpretation of our findings. Therefore, it’s of the essence to confirm whether this correlation holds in a larger HF patient cohort. Furthermore, due to the limited sample size, we were unable to construct a comprehensive model encompassing multiple parameters. Second, this current study did not focus on HF patients, therefore, circumstances such as elevated PCWP or atrial tachyarrhythmia, often coincide with HF, were underrepresented, potentially biasing the study outcomes. Additionally, exclusively patients with severe aortic stenosis were included in this study. Limited data demonstrated that this likely influences the PPG-waveform by increasing the probability of a smooth dicrotic notch.^11^ Nonetheless, similar waveform features have been observed in various other cardiovascular diseases in the same study. Future research should aim to clarify the specific impact of different cardiovascular conditions on waveforms, thereby improving interpretability and applicability of PPG data in a clinical setting. Third, in the current study, a cut-off of 12 mmHg was employed as the upper limit for a physiologically normal PCWP. However, a higher cut-off, specifically 15 mmHg, is considered more clinically relevant within the HF population. Unfortunately, due to the limited number of patients with a PCWP above 15 mmHg, we were unable to construct models using this higher threshold. Fourth, time periods for PPG analysis were manually selected based on signal quality that potentially introduces selection bias. Given inherent noise in wearable PPG data, determining requisite data volume for accurate PCWP estimation is pivotal. Last, it should be noted that PCWP alone is an imperfect reference standard for haemodynamic congestion, embracing additional parameters relevant to HF could be crucial for comprehensive patient evaluation Although encouraging, our pilot study mandates further research to formulate and validate a PPG-based model before contemplating testing within a substantial prospective trial.

## Conclusion

In this pilot study, we demonstrated the capability to non-invasively classify patients into clinically meaningful categories of PCWP using PPG signals from a wrist-worn wearable device. While we acknowledge the limitations of a pilot study, these initial results highlight the promising nature of PPG-based non-invasive haemodynamic monitoring. Subsequent larger studies involving this technology within HF populations are necessary to further refine this model and explore its potential applications.

## Data Availability

The data underlying this article will be shared on reasonable request to the corresponding author.
